# Benefits of Older Volunteering on Wellbeing: An International Comparison

**DOI:** 10.3389/fpsyg.2019.02647

**Published:** 2019-12-13

**Authors:** Marta Gil-Lacruz, María I. Saz-Gil, Ana I. Gil-Lacruz

**Affiliations:** ^1^Department of Psychology and Sociology, Faculty of Economics and Business, University of Zaragoza, Zaragoza, Spain; ^2^Department of Business Management and Organization, Faculty of Economics and Business, University of Zaragoza, Zaragoza, Spain

**Keywords:** volunteering, non-profit organizations, aging, lifestyles, wellbeing, health

## Abstract

Healthier aging implies lower health service expenditure and the possibility for individuals to make a longer and more valuable contribution to society. Lifestyles, including volunteering, affect our health. The policy implications of the present study are that it aims to broaden the state of knowledge and be useful to public decision-makers: if voluntary activities enhance the integration of older people into society, their participation will help to generate economic resources and improve their own welfare; if, however, health and participation do not show positive synergies, then policymakers must act independently in each of these fields. In this work, we focus on the societies of Chile, Mexico, and Spain because they have significantly aging populations and share common traits but also exhibit important differences. The empirical study employs micro-data from the World Value Survey (1994–1998, 2005–2009, and 2010–2014) and macro-data from the statistics of the OECD (Organization for Economic Co-operation and Development). Micro- and macro-data are merged by national and temporal identifiers. Our main results provide empirical evidence that volunteering might improve every indicator of wellbeing except happiness. Different kinds of activities have different impacts on individual wellbeing. For example, volunteering in activities related to social awareness is positive for male life satisfaction, whereas volunteering in activities related to religious issues is positive for male life satisfaction but also female happiness. In general, voluntary activities have a stronger impact on male wellbeing than female wellbeing.

## Introduction

One of our major public health challenges is to achieve healthy aging: if the necessary measures are not taken, the reversal of the population pyramid will have a huge impact on the economic, social development, and healthcare systems (WHO, 2012). Quality of life depends on multiple factors such as access to and use of education and health services, biological processes, and environmental impact but also on individual behaviors and socioeconomic characteristics (Urzúa et al., 2011; Salvador-Carulla et al., 2014). It is necessary to promote health throughout the lifecycle so that people may enjoy the best possible wellbeing in old age (Pinquart and Sörensen, 2000; Brett et al., 2012).

Lifestyles that include physical inactivity, a high-fat diet, or tobacco and alcohol consumption have frequently been studied by the scientific community as behaviors that influence the state of health in older adults (Vagetti et al., 2015). It seems that retired and senior volunteers are more protected from the hazards of retirement, physical decline, and inactivity than people of the same age who do not perform volunteer work (Fischer and Schaffer, 1993; Wilson and Musick, 1999). The main aim of this paper is to study the social determinants of the wellbeing of older adults, with particular emphasis on volunteering. The study of the link between volunteering and subjective wellbeing is not new, but only a few studies have covered a broader range of such activities performed specifically by older adults (Ferring and Boll, 2010). In addition, while the positive correlation between volunteering and wellbeing is widely documented, there are many unanswered questions concerning omitted variables, bias, self-selection, and reverse causality (Stukas et al., 2016).

The main contribution of this article is to provide an international analysis of the correlations of three indicators of wellbeing with four categories of voluntarism. We repeat estimations by sub-samples of men and women in order to account for gender differences.

Existing research has typically adopted a global measure of volunteering without differentiating between different volunteering activities (Cheng and Chan, 2018). It is important to control for different types of voluntary activities, because, for example, other-oriented volunteers may experience greater benefits to their health, as they get higher satisfaction from the volunteering than self-oriented volunteers (Stukas et al., 2016). In our analysis, as well as the variable that informs us whether the individual is a volunteer, other variables examine voluntary activities classified as religious, social awareness, professional and political, and/or education and leisure. Whereas the first two types of volunteering might have intrinsic motivations, the last two might be characterized by extrinsic motivations (Sardinha, 2010).

Regarding wellbeing, we have measured older adults’ wellbeing with self-reported values of health, happiness, and life satisfaction. Life satisfaction and happiness are not only important indicators of wellbeing; they also have a strong effect on an individual’s self-reported state of health (Ruiz-Aranda et al., 2014). Nevertheless, we consider it pertinent to include three indicators of wellbeing because volunteering might determine each wellbeing domain in a different way (Binder, 2015). For instance, being a volunteer in social awareness activities might have a positive impact on life satisfaction but a negative effect on happiness. Lastly, complementing traditional objective indicators of quality of life (such as economic indicators) with indicators of subjective wellbeing (thus, people’s evaluations and feelings about their lives) will provide policymakers with more relevant information for political decision-making and public program evaluation (Diener et al., 2008).

Concerning the international dimension, this article focuses on Chile, Mexico, and Spain for two main reasons:

(i) Spanish society and Latin American societies are aging rapidly, and elderly people are an important population group. Spain and Chile are in an advanced state of aging, with a natural population growth^1^ of 0.1 and 0.9%, respectively (the rate of natural population growth is the birth rate minus the death rate, expressed as a percentage). Mexico is also in a full aging stage with a rate of 1.4% (Population Reference Bureau, 2014). In this context, welfare systems have to support a larger population that does not financially contribute, or at least its contributions are not valued in the national accounts. Although unpaid voluntary work is often ignored in national accounts, the economic contribution of volunteers is important, and it is therefore necessary to quantify it and make it visible.
(ii) In addition to the common impact of globalization, Spain and Latin American countries share similarities (for example, their health care systems and cultural backgrounds) that facilitate comparison. A recent study based on the healthcare costs and inflation (among other indices) of 51 countries put the healthcare systems of Mexico, Spain, and Chile in 12th, 14th, and 17th positions, respectively (Bloomberg, 2014). Life expectancy is higher in Spain (82 years) than in Chile (79 years) and Mexico (77 years), but it is increasing in Chile and Mexico and falling in Spain. The percentage of the GDP allocated to healthcare is similar in the three countries; the nominal value is much greater in Spain ($2.808) than in Chile ($1.103) and Mexico ($618).

In the last 15 years, both Chile and Mexico have invested in public healthcare in order to cover a greater section of the population. Chilean healthcare is close to universal, although health indicators still vary greatly across socioeconomic groups, reflecting economic inequality (Bunout et al., 2012). Low-income populations are mainly covered by the public sector, while high-income populations generally turn to the private sector (Vargas and Poblete, 2008; Missoni and Solimano, 2010). In Mexico, there has been a major legislative reform that is increasing healthcare funding by 1% of the 2003 gross domestic product over 7 years to provide universal health insurance. Results indicate significant progress in public healthcare (Frenk et al., 2009). In this context, the quality of life of older adults is becoming a new research topic in relation to healthy and active aging (Wong et al., 2010).

In contrast, after suffering a severe economic crisis, the Spanish government has made severecuts to its social and healthcare services: universal health coverage has ended, and changes have been made to health insurance, benefits, and funding (Gallo and Gené-Badía, 2013).

This article does not aim to be a cross-cultural study where the main point is to address cultural differences (Ilesanmi, 2009), though we do expect to identify national differences and similarities. Given that sub-samples by country might differ at the individual level (micro-data: surveys) but also in information from primary sources (macro-data: economic indicators), the policy implications of our results will benefit from the introduction of national economic statistics on public expenditures and average wages.

In this situation, we carry out research on older adults with two dimensions: on the one hand, an analysis of the socioeconomic determinants of volunteering, and on the other, a study of the link between wellbeing and volunteering activities. Gender and cultural differences are expected, because, in spite of sharing common backgrounds, Mexico, Chile, and Spain have different socioeconomic contexts.

### Literature Review

Subjective wellbeing refers to an evaluation of an individual’s life from their own perspective, which contrasts with evaluations made from the point of view of external observers (including researchers and policymakers), which are based on objective criteria (Ferring and Boll, 2010). Even if we only focus on subjective wellbeing, it can be assessed with measures of distinct concepts such as satisfaction with life, happiness, quality of life, and life fulfillment (Bartels and Boomsma, 2009). In addition, not only is how to conceptualize subjective wellbeing a key issue for health researchers but so also is how to introduce it into empirical strategies. The literature regarding subjective wellbeing treats it as an effect, as the result of personal and environmental variables, but also as a predictor, mediator, and moderator of other outcomes (Ferring and Boll, 2010).

In this article, we relate the wellbeing of senior volunteers with their volunteering. Voluntary work provides a wide range of benefits to the volunteer but also to the social group, community, and society where this behavior takes place. It has been shown that the volunteers themselves can share in the positive effects of their actions (Taylor, 2004; Ronel, 2006; Amendola et al., 2011; Binder and Freytag, 2013). Given the positive influence of civil and community associational activities, policy-makers should promote the development and consolidation of high levels of social capital (Sabatini, 2008). The design of strategies to encourage volunteering among older people requires prior knowledge of the characteristics of the older population. Volunteering needs to be perceived as an altruistic activity that also benefits the volunteers (Fiorillo and Sabatini, 2011). There is empirical evidence that retired and senior volunteering has a positive effect on all the stakeholders: the voluntary organizations (NGOs), the people they serve, and the volunteers (Mannino et al., 2011).

Studies have shown that volunteers report a better state of health and wellbeing than non-volunteers (Stephan, 1991; Moen et al., 1992; Sabin, 1993; Rogers, 1996; Musick et al., 1999; Oman et al., 1999; Borgonovi, 2008) and that they even have a longer life expectancy (Harlow and Cantor, 1996; Okun et al., 2010; Burr et al., 2011; Fothergill et al., 2011). Recent research suggests that participating in volunteer activities during leisure time positively reinforces health and happiness (Post, 2005; Guven, 2011; Harvey, 2011). A longitudinal study for retired men and women revealed that the frequency of volunteering, as well as future intentions to perform such activities, predicted higher positive affect in the feeling of ability (Pushkar et al., 2010). One of the scarce studies with panel data found positive evidence for the moderating influence of volunteering on the relationship between negative self-esteem and wellbeing (Russell et al., 2019). The results of these articles suggest that volunteering might act as a safeguard for seniors.

Nevertheless, older people vary greatly in their health, financial resources, and social networks and should not be seen as a homogenous group. Senior volunteers should only contribute in the way in which they feel comfortable, and no expectations beyond their capabilities should be placed on them by organizations (Stephens et al., 2015). Under this premise, even for older adults with multiple chronic diseases, participating in volunteer activities seems to improve their self-reported state of health (Barron et al., 2009).

The intrinsic motivations for volunteering are usually taken as a starting point for identifying volunteer profiles. The motivation to the volunteer may be relevant for inferring individual characteristics that encourage specific behaviors. Some personality traits (for example, extraversion) influence volunteering decisions and life satisfaction (Cocca-Bates and Neal- Boylan, 2011; Kwok et al., 2013). Motivations are social constructs with important gender differences. Women score higher on traits, motivations, and values that predict informal help-care (family and friends). Men have more resources (income, education, and social capital), which compensates for their lower level of motivation. Therefore, gender gaps in the institutional helping behaviors of volunteering and charitable giving are small (Einolf, 2011).

Among other factors, the sense of belonging to a wider community plays a key role in older adults’ decisions on volunteering (Sarason, 1986; Pozzi et al., 2014). Retired and senior volunteering could be highly beneficial for societies with an aging population; participating in voluntary activities can empower older people, mitigating the difficulties of retirement, physical decline, and inactivity. Older adult volunteering can prevent social isolation (a major risk factor for mortality among the elderly) and help maintain and even improve mental health (House et al., 1988; Ahern and Hendryx, 2008; Chen, 2013).

The majority of retired and senior volunteers participate because they have been asked, although a minority actively seeks voluntary opportunities (Chen and Morrow-Howell, 2015). Seniors moving into retirement choose volunteering as a way to participate in meaningful activities while maintaining their personal beliefs and social ties. Change predisposition and self-transcendence are values that could be involved in this behavior by elders (Ariza-Montes et al., 2017). However, the factors that influence how seniors choose to volunteer in a community are not well understood. Undoubtedly, knowing the determinants of volunteering involvement will help the recruitment of senior volunteers (Heist et al., 2019).

Social relationships are important, but when analyzing their instrumental value, it is essential to determine whether such relationships are perceived as ‘satisfactory’ or not. Processes and networks of mutual assistance, dissemination of information relating to health, and promotion of healthy behaviors only develop in contexts of high-quality relationships (Fiorillo and Sabatini, 2011). For example, different types of volunteering activities require different volunteer profiles. Volunteering in a professional organization is not the same as in a religious group (Gil-Lacruz and Marcuello, 2013). Understanding the different kinds of voluntary activities might be especially relevant when analyzing the impact of volunteering on older adults’ wellbeing.

Wellbeing is profoundly affected by who we are and where we live (Doyal, 2000). We have to take the issue of health diversity very seriously (Wei et al., 2006), especially when advocating effective international policies on equity and human rights. The impact of social relationships and support networks on wellbeing depends on a variety of demographic and environmental factors such as age, employment, residence (urban or rural), and genetic resilience (Moss, 2002). Volunteering is associated with a social gradient, with disadvantaged groups less likely to volunteer. Given the potential benefits of volunteering for all stakeholders, volunteering should be considered as a public health strategy to tackle social exclusion and health inequalities (South et al., 2016).

## Materials and Methods

The World Values Survey (WVS; World Values Survey Association, 2017) is a powerful resource for the study of the socioeconomic determinants of wellbeing. At this time, there are seven available waves, but, for this study, we used three: for the periods 1994–1998, 2005–2009, and 2010–2014. The main reason why we limited the empirical analysis to these three waves was to ensure that the questions included in the questionnaires were comparable over time: the three aforementioned waves contain data for Chile, Mexico, and Spain. This research is based on older adults from 61 to 80 years old. Our sample is composed of 1,699 observations, of which 24% correspond to Chile (798), 48% to Mexico (901), and the remaining 28% to Spain (476).

The macro variables have been taken from OECD (Organization for Economic Co-operation and Development) health statistics. We have taken into account national wage rates and public expenditure on health, old age, and other similar issues. International comparisons are facilitated by measuring the variables in per capita terms, adjusted to purchasing power parity, and given in US dollars.

The data used for this research was obtained from public databases available on the internet^1^ and from pre-existing publications^2^.

### Measures

We have included the following explanatory variables: sex (*Men* and *Women*), age in four groups (*Age: 61–65*, *Age: 66–70*, *Age: 71–75*, and *Age: 76–80*), marital status (*Married*, *Single*, *Divorced*, and *Widow/Widower*), labor situation (*Retired*, *Housewife*, *Unemployed*, and *Working*), highest educational level achieved (*Primary Studies*: typically the first stage of formal education, coming after preschool and before secondary education, *Secondary Studies*: considered the second and final phase of basic education and is the stage before tertiary education, and *Tertiary Studies*: the educational level including universities as well as trade schools and colleges), number of children living in the same household (*Number of Children*), and level of income (*Low Income*: population tertile with the lowest income level in the corresponding country, *Middle Income*: population tertile with the second-lowest income level, and *High Income*: population tertile with the highest income level).

Most empirical studies on the subjective wellbeing of older adults have relied on self-reports obtained in questionnaires or interviews (Ferring and Boll, 2010). As indicators of the wellbeing of older adults in these countries, we considered self-reported health, happiness, and life satisfaction on a scale of 1 (excellent) to 5 (very poor). We defined three dummy variables for the categories: ‘1’ denotes that the individual has excellent or very good levels and ‘0’ indicates otherwise.

Because most of these variables are dummy variables (1/0), their means as percentages (multiplying by 100) give us their corresponding participant distribution. For example, the mean of *GoodHealth* is 0.44, which means that 44% of the participants reported good health. Table 1 shows that the total sample is equally distributed between men (47%) and women (53%), with small national variations for the percentage of women in Chile (57%), Mexico (49%), and Spain (55%). There is an over-representation of the younger cohorts (from 61 to 65 years old), and this is more significant in Chile (37%) and Mexico (44%). The majority of the senior citizens are married (65%), and this is especially true in Spain (73%). There are more widows and widowers in Chile (30%). There are many more men (66%) who are retired than women (36%), and more Spaniards (67%) are retired than Chileans (51%) or Mexicans (29%). There are more male seniors (25%) working than female seniors (9%), and older Chileans (23%) and Mexicans (29%) are working than Spaniards (8%). Not surprisingly, many more women (53%) are homemakers than men (1%). Most of the retired and senior citizens have primary studies or no studies at all (78%); Chileans (64%) and Mexicans (71%) have better levels of education than Spaniards (88%). Income levels by tertiles are provided by the WVS. Taking into account that our observations correspond to a sub-sample of the whole data, older adults reported worse economic conditions than the general population: 52% of them reported low income, 43% middle income, and only 5% high income.

**TABLE 1:** Variables (means by general category, gender, and country).

	**World value survey**					
	
	**Total**	**Women**	**Men**	**Chile**	**Mexico**	**Spain**
	**%**	**%**	**%**	**%**	**%**	**%**
GoodHealth	44	40	49	36	44	53
Happiness	81	77	85	76	88	85
LifeSatisfaction	63	59	67	65	87	55
UnpaidWork	40	42	39	41	61	22
UnpaidSocialAwareness	9	9	10	5	19	5
UnpaidProfessional	8	6	10	3	20	3
UnpaidEducationLeisure	10	7	13	8	15	7
UnpaidReligion	29	35	23	34	45	12
Men	47	–	100	43	51	45
Women	53	100	–	57	49	55
Age: 61–65	36	36	36	37	44	35
Age: 66–70	31	30	31	33	28	29
Age: 71–75	20	19	22	18	21	20
Age: 76–80	13	15	11	12	7	16
Married	65	54	77	56	61	73
Single	6	7	4	7	6	5
Divorced	5	6	4	7	9	3
Widow/widower	24	33	15	30	24	19
Retired	52	36	66	51	29	67
Housewife	28	53	1	26	29	24
Unemployed	4	2	8	1	13	2
Working	16	9	25	23	29	8
Primary Studies	78	81	75	64	71	88
Secondary Studies	16	15	17	30	18	8
Tertiary Studies	6	4	8	6	11	3
Number of Children	3.3	3.3	3.4	3.3	4.6	2.6
Low Income	52	54	49	44	59	50
Middle Income	43	42	45	50	30	48
High Income	5	4	6	6	11	2
Number of observations:	1,699	901	798	408	815	476

	**OECD health data**				
	
	**Chile**	**Mexico**	**Spain**
Public Health Expenditure	434	360	1,596
Public Old Age Expenditure	495	163	2,110
Other Public Expenditure	556	416	2,473
National Wage	15,347	13,168	26,648

*We have also included Regional Dummy Variables (Spain, Mexico, and Chile) and Time Dummy Variables (Wave: 1994–1998, Wave: 2005–2009, and Wave: 2010–2014). Data from the corresponding National Surveys, Years: Chile (1996, 2006, and 2011), Mexico (1995/96, 2005, and 2012), and Spain (1995, 2007, and 2011).*

**FIGURE 1:**
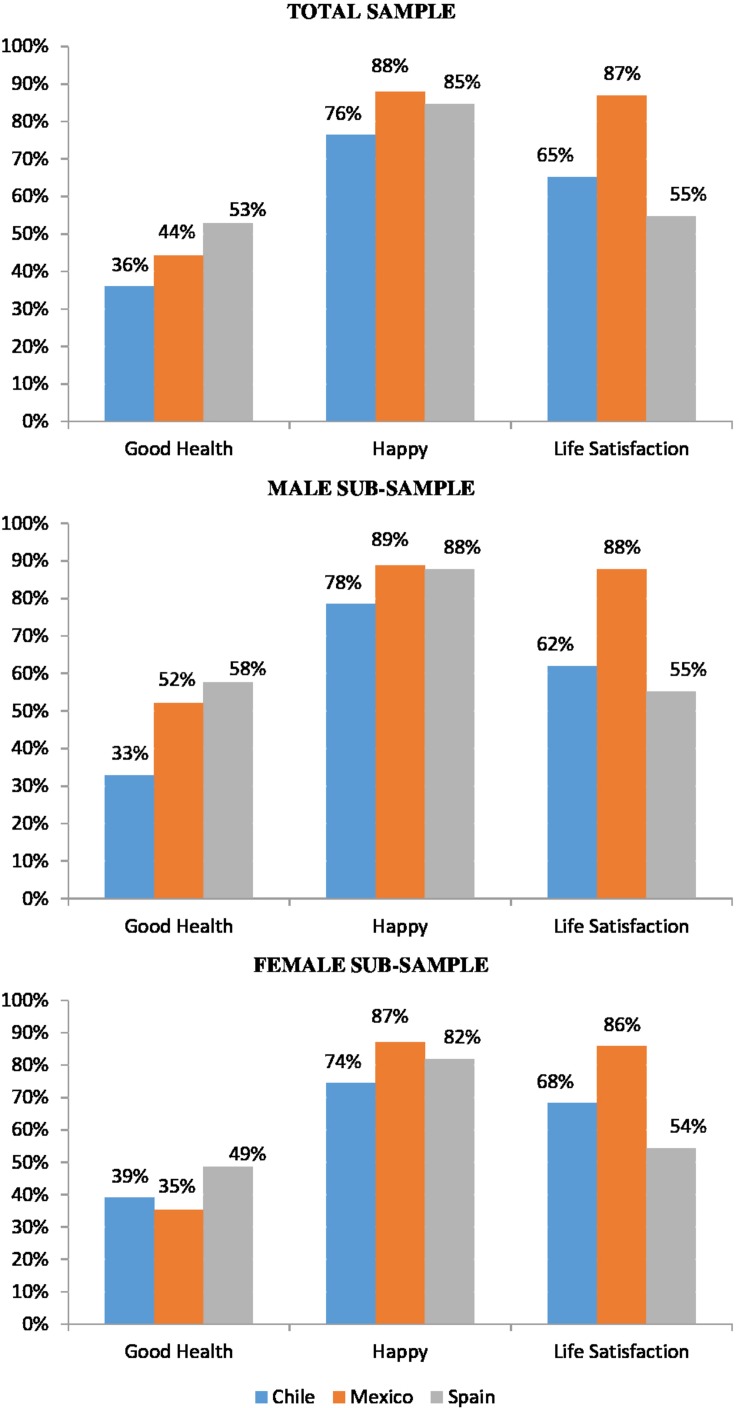
Health and wellbeing indicators (Wave: 2010–2014).

Figure 1 shows the levels of health, happiness, and life satisfaction of the older adults by country of residence. Only half of elderly Spaniards believe they have a good state of health, followed by Mexicans (44%) and Chileans (36%). Nevertheless, levels of happiness are high: the vast majority of older Mexicans (88%), Spaniards (85%), and Chileans (76%) say that they are happy or very happy. The biggest geographical gap was found with regards to life satisfaction: almost 90% of Mexicans give a very high value to their level of life satisfaction, but this figure is only 65% in Chile and 55% in Spain. Gender differences show that elderly women from Mexico and Spain report a worse state of health but a higher level of life satisfaction than men, but the reverse is true for the older adults in Chile.

**FIGURE 2:**
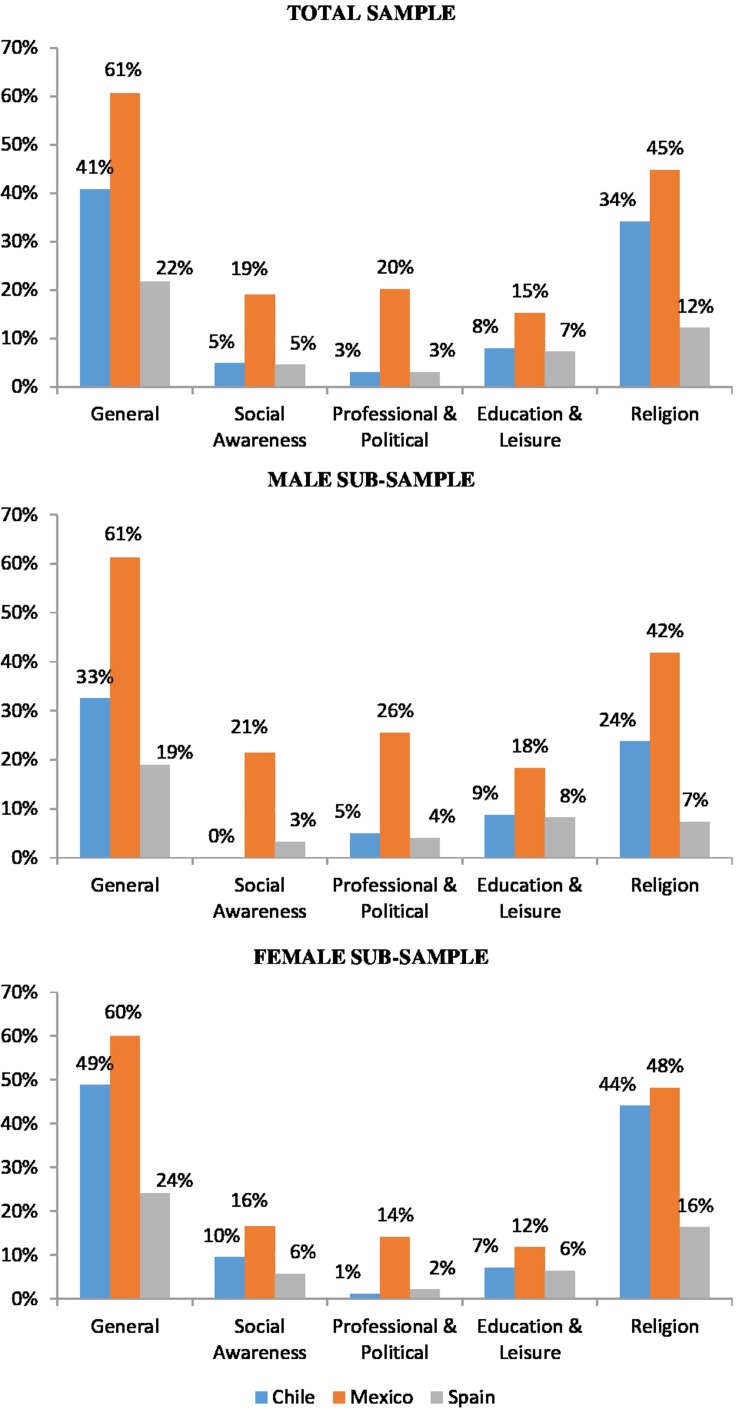
Volunteers: general percentage and by categories (Wave: 2010–2014).

The WVS includes data on voluntary participation in 11 different volunteering activities. Keeping all these categories would have implied considering 11 dummy variables, which would have prolonged the extension of the tables, with less powerful results (some categories have low participation rates). According to Sardinha (2010), the different types of volunteering activities included by the WVS can be distributed into four categories: (1) Social awareness: activities related to human rights or environmental and animal conservation; (2) Professional and Political: activities related to trade unions, political parties, and professional associations; (3) Education and Leisure: activities related to education, culture, youth work, sports, or leisure; (4) Religion: activities in churches or religious organizations. The rewards of volunteering depend on the volunteers’ motivation, which might be basically classified as intrinsic motivation and extrinsic motivation (Meier and Stutzer, 2004). Volunteering in the fields of social awareness and religion might be motivated by a desire to help others (intrinsic); the motivation for volunteering in professional and leisure activities might be more personal, involving self-interest (extrinsic). Figure 2 shows participation rates of different volunteering activities by gender in the sample.

Retired and senior Mexicans are the most likely to volunteer (61%), followed by Chileans (41%) and Spaniards (22%). The most common category area for voluntary work is religion, followed by professional and political activities, social awareness activities, and education and leisure activities. Mexicans report the highest rates for volunteering in the different categories. Older women are more likely to volunteer in activities related to religion, whilst older men are more likely to volunteer in activities related to education and leisure and on professional and political issues.

### Procedure

As the main aim of this study was to analyze the determinants of the wellbeing of retired and older adults, we used an empirical framework that estimated qualitative dependent variables (1: yes; 0: otherwise). Estimation techniques depend on the nature of the data used (cross-section) and the aims (estimated probabilities). We focused on Logit models, reporting results in terms of elasticities. Our empirical framework is similar to that used in previous econometric studies that employ cross-sectional data to identify the causal effects of health behaviors (Gil-Lacruz et al., 2015; Estrada-Fernandez et al., 2017):

(1) $$H_{j\,i}=V_{j\,i}\,\delta_{j}+X_{j\,i}\,\beta_{j}+M_{j\,i}\,\lambda_{j}+C_{j\,i}\,\alpha_{j}+u_{j\,i}$$

*H* is the self-perceived indicator of wellbeing. *V* indicates whether the individual is involved in voluntary activity. *X* is a vector of sociodemographic characteristics (age, gender, civil status, number of children, employment, educative level, and economic status). *M* are macro variables (average national salary and public expenditure on health, old age, and other issues). Macro variables are introduced in Napierian logarithms. They are relevant due to the information they provide, and they also reduce unobserved heterogeneity caused by national backgrounds. The estimated parameters of geographical dummy variables are high in magnitude but not especially relevant in terms of statistical significance. After controlling for a relevant set of explanatory variables, we have not found a relevant country variation, even when implementing a multilevel analysis to check whether there is a hierarchical structure at the country level. *C* are contextual variables (country of residence and survey year), and *u* is an error term whose mean is equal to zero. The sub-index *i* identifies the interviewed individuals, and the sub-index *j* takes the value 1 for Good Health, 2 for Happiness, and 3 for Life Satisfaction.

We have considered three independent models: in Model 1, we did not include voluntary activity in order to evaluate the robustness of the parameters of the other explanatory variables; in Model 2, we considered voluntary activity in one category (*UnpaidWork*); in Model 3, we considered four types of voluntary activities (*UnpaidSocialAwareness*, *UnpaidProfessional*, *UnpaidEducationLeisure*, and *UnpaidReligion*).

We were aware of potential endogeneity problems and carried out the Schmith-Blundell exogeneity test; we found no empirical evidence of strong endogeneity problems. Given the fact that the magnitude of the estimated parameters of voluntary activities was not especially high and that the rest of the estimated parameters remained robust through the models, we decided to keep the empirical strategy as simple as possible.

Estimations were repeated by gender subsamples to check whether gender gaps were based on differences in the composition of explanatory variables or on diverse reactions to similar stimuli.

To analyze the social determinants of wellbeing among older adults, we focus on the following hypotheses:

H1: There is a positive association between volunteering and wellbeing.
H2: The association between volunteering and wellbeing depends on the type of volunteering activity.
H3: Gender is an important explanatory factor. Besides, sub-samples of men and women help to understand gender differences on wellbeing, so gender gaps might be based on different individual characteristics (endowment effects) and/or on the different impacts of these characteristics (coefficient effects).
H4: Country dummy variables remain as important determinants for wellbeing after controlling for a wide set of variables.

## Results

This section is devoted to the determinants of three indicators of wellbeing (health, happiness, and life satisfaction). The numbers in parentheses represent the estimated coefficients of the elasticities. The term elasticity (dy/dx) refers to a measure of the dependent variable’s sensitivity to a change in an explanatory variable. For the individual significance of parameters, we add stars to the estimated coefficients, thus, ^∗∗∗^ reflects a significance level of 1% (*p*-value ≤ 0.01), ^∗∗^ a significance of 5% (0.01 < *p*-value ≤ 0.05), and ^∗^ a significance of 10% (0.05 < *p*-value ≤ 0.1). For global goodness of fit, we take into account the pseudo-R2, which summarizes the proportion of variance in the dependent variable associated with the explanatory variables (a larger pseudo-*R*^2^ value indicates that more of the variation is explained by the model, to a maximum of 1) and estimated probabilities of dependent variables (the closer the estimated value is to the real, the better).

The results of Model 1 in Table 2 reveal that women perceive their state of health (dy/dx: −0.078^∗∗^) as worse than do men (variable of reference) and they are also less happy (dy/dx: −0.046^∗^). People aged from 61 to 65 are less satisfied with life (dy/dx: −0.058^∗^) than individuals aged from 66 to 70 years old (variable of reference). Being single (dy/dx: −0.123^∗∗∗^), divorced (dy/dx: −0.168^∗∗∗^), or a widow/widower (dy/dx: –0.114^∗∗∗^) has a negative effect on the level of happiness versus being married (variable of reference); being divorced (dy/dx: −0.147^∗∗∗^) or a widow/widower (dy/dx: −0.080^∗∗∗^) has a negative effect on the level of life satisfaction. Older people that are unemployed (dy/dx: 0.141^∗∗^) or working (dy/dx: 0.066^∗^) perceive themselves as healthier than people that have retired (variable of reference). Higher levels of education are associated with better health (dy/dx: 0.122^∗∗∗^ and 0.180^∗∗∗^ for secondary studies and tertiary studies versus primary studies, which is the reference variable), greater happiness (dy/dx: 0.096^∗∗∗^ and 0.119^∗^ for secondary studies and tertiary studies), and improved life satisfaction (dy/dx: 0.075^∗∗^ and 0.156^∗∗^ for secondary studies and tertiary studies). Higher levels of income are also associated with better health (dy/dx: 0.118^∗∗∗^ and 0.197^∗∗∗^ for middle income and high income versus low income, which is the reference variable), greater happiness (dy/dx: 0.092^∗∗∗^ for middle income), and improved life satisfaction (dy/dx: 0.115^∗∗∗^ for middle income).

**TABLE 2:** Estimations of wellbeing indicators: Model 1 (Logit: dy/dx).

	**Good health**	**Happiness**	**Life satisfaction**
Men^a^	–	–	–
Women	–0.078^∗∗^	−0.046^∗^	–0.029
Age: 61–65	0.042	–0.018	−0.058^∗^
Age: 66–70	–	–	–
Age: 71–75	0.013	–0.008	–0.023
Age: 76–80	–0.037	0.003	0.023
Married^a^	–	–	–
Single	0.043	–0.123^∗∗∗^	0.034
Divorced	–0.013	–0.168^∗∗∗^	–0.147^∗∗∗^
Widow/widower	–0.029	–0.114^∗∗∗^	–0.080^∗∗∗^
Retired^a^	–	–	–
Housewife	0.041	0.002	0.011
Unemployed	0.141^∗∗^	0.017	0.075
Working	0.066^∗^	0.018	0.049
Primary Studies^a^	–	–	–
Secondary Studies	0.122^∗∗∗^	0.096^∗∗∗^	0.075^∗∗^
Tertiary Studies	0.180^∗∗∗^	0.119^∗^	0.156^∗∗^
Number of Children	−0.012^∗^	–0.003	0.006
Low Income^a^	–	–	–
Middle Income	0.118^∗∗∗^	0.092^∗∗∗^	0.115^∗∗∗^
High Income	0.197^∗∗∗^	0.077	0.118^∗^
Ln(Public Health Expenditure)	−0.864^∗^	–0.478	−0.898^∗^
Ln(Public Old Age Expenditure)	0.182^∗∗∗^	0.125^∗∗∗^	0.036
Ln(Other Public Expenditure)	–0.073	0.329^∗∗^	0.118
Ln(National Wage)	0.674^∗^	0.385	1.124^∗∗∗^
Spain^a^	–	–	–
Mexico	−0.607^∗^	0.503^∗∗^	0.080
Chile	–0.756^∗∗^	0.169	–0.268
Wave: 1994–1998^a^	–	–	–
Wave: 2005–2009	–0.071	–0.196	–0.350^∗∗^
Wave: 2010–2014	–0.031	−0.274^∗^	–0.390^∗∗^
Pseudo *R*^2^	6%	13%	10%
Estimated probability	46%	81%	63%

*^*a*^Reference variable. ^∗∗∗^, ^∗∗^, and ^∗^ indicate that the explanatory variables are significant at 1, 5, and 10%.*

Turning to the macro variables, a higher average national salary is associated with better levels of health (dy/dx: 0.674^∗^) and life satisfaction (dy/dx: 1.124^∗∗∗^). The degree of public expenditure on health does not have a positive impact on the indicators of wellbeing (dy/dx: −0.864^∗^ for good health and dy/dx: −0.898^∗^ for life satisfaction), but public expenditure on issues concerning old age increases levels of health (dy/dx: 0.182^∗∗∗^) and happiness (dy/dx: 0.125^∗∗∗^).

When controlled for socioeconomic determinants, Spaniards (the reference variable) are more likely to have a good state of health than Mexicans (dy/dx: −0.607^∗^) or Chileans (dy/dx: −0.756^∗∗^); however, Mexicans (dy/dx: 0.503^∗∗^) are more likely to be happy than Spaniards. Levels of happiness (dy/dx: −0.274^∗^ for Wave: 2010–2014 versus Wave: 1994–1998, which is the reference variable) and life satisfaction (dy/dx: −0.350^∗∗^ and −0.390^∗∗^ for Wave: 2010–2014 and Wave: 2005–2009) become lower over time.

Model 2 and Model 3 included variables related to voluntary activities. Although both models share the explanatory variables of Model 1, their estimations were carried out independently. The estimated parameters of the socioeconomic determinants of Model 1 remained robust so, to avoid repeating the same results and to facilitate comparisons between Model 2 and Model 3, we present the most characteristic results of both models in a single table. The same comment is valid for Tables 3, 5. Regarding the particular information in Table 3, being a volunteer improves perceived health (dy/dx: 0.062^∗∗^) and life satisfaction (dy/dx: 0.052^∗∗^). Being a volunteer in activities related to social awareness has a negative impact on happiness (dy/dx: −0.103^∗∗∗^); religion-based voluntary activity improves life satisfaction (dy/dx: 0.061^∗∗^).

**TABLE 3:** Estimations of wellbeing indicators: Model 2 and Model 3 (Logit: dy/dx).

	**Good**	**Happiness**	**Life**
	**health**		**satisfaction**
**Model 2^a^**			
UnpaidWork	0.062^∗∗^	–0.007	0.052^∗∗^
Pseudo *R*^2^	7%	13%	10%
Estimated probability	46%	81%	63%
**Endogeneity:** Instrument (national percentage affiliated to NGOs)			
Schmith-Bundell exogeneity test	No problem	No problem	No problem
**Model 3^a^**			
UnpaidSocialAwareness	0.011	–0.103^∗∗∗^	0.080
UnpaidProfessional	0.021	0.001	0.012
UnpaidEducationLeisure	0.042	0.056	0.010
UnpaidReligion	0.037	–0.005	0.061^∗∗^
Pseudo *R*^2^	6%	13%	11%
Estimated probability	46%	81%	63%
**Endogeneity:** Instrument (national percentages affiliated to NGOs for each category)			
Schmith-Bundell exogeneity test	No problem	No problem	No problem

*^*a*^We have included explanatory variables of Model 1. Estimated coefficients are robust, so we have omitted their transcription. Results are available on request to authors. ^∗∗∗^ and ^∗∗^, indicate that the explanatory variables are significant at 1 and 5%.*

**TABLE 4:** Estimations of wellbeing indicators: Model 1 by gender (Logit: dy/dx).

	**Women**			**Men**		
		
	**Good health**	**Happiness**	**Life satisfaction**	**Good health**	**Happiness**	**Life satisfaction**
Age: 61–65	0.077^∗^	–0.045	−0.076^∗^	0.011	0.007	–0.049
Age: 66–70^a^	–	–	–	–	–	–
Age: 71–75	–0.024	−0.073^∗^	–0.018	0.043	0.073^∗^	–0.029
Age: 76–80	–0.057	–0.022	0.011	0.005	0.027	0.032
Married^a^	–	–	–	–	–	–
Single	0.003	–0.173^∗∗∗^	0.070	0.098	–0.089	–0.022
Divorced	–0.040	–0.225^∗∗∗^	−0.141^∗^	–0.019	–0.120^∗∗∗^	–0.165^∗∗^
Widow/widower	–0.026	–0.177^∗∗∗^	–0.090^∗∗^	0.009	–0.035	–0.068
Retired^a^	–	–	–	–	–	–
Housewife	0.040	–0.027	0.008	0.362	(omitted)	(omitted)
Unemployed	0.326^∗∗^	–0.136	–0.149	0.110	0.072	0.160^∗^
Working	0.073	0.007	0.074	0.089^∗^	0.026	0.037
Primary Studies^a^	–	–	–	–	–	–
Secondary Studies	0.186^∗∗∗^	0.071	0.117^∗∗^	0.074	0.120^∗∗∗^	0.035
Tertiary Studies	0.224^∗∗^	0.081	0.155	0.179^∗∗^	0.161^∗∗^	0.173^∗∗^
Number of Children	–0.011	–0.012	0.005	–0.010	0.003	0.006
Low Income^a^	–	–	–	–	–	–
Middle Income	0.128^∗∗∗^	0.109^∗∗∗^	0.101^∗∗∗^	0.110^∗∗∗^	0.069^∗∗^	0.123^∗∗∗^
High Income	0.295^∗∗∗^	0.075	0.134	0.162^∗^	0.072	0.086
Ln(Public Health Expenditure)	–0.619	–0.300	–1.109	–0.868	–0.493	–0.511
Ln(Public Old Age Expenditure)	0.087	0.133^∗∗^	0.018	0.231^∗∗∗^	0.129^∗∗^	0.049
Ln(Other Public Expenditure)	–0.433	0.329	0.271	0.000	0.340^∗∗^	–0.043
Ln(National Wage)	0.786	0.264	1.420^∗∗^	0.497	0.326	0.627
Spain^a^	–	–	–	–	–	–
Mexico	–1.069^∗∗^	0.758^∗∗^	0.217	–0.491	0.404	0.014
Chile	–1.051^∗∗^	0.336	–0.172	–0.722	0.144	–0.264
Wave: 1994–1998^a^	–	–	–	–	–	–
Wave: 2005–2009	–0.141	–0.148	–0.516^∗∗^	0.075	–0.213	–0.119
Wave: 2010–2014	0.006	–0.228	–0.542^∗∗^	0.047	–0.303	–0.180
Pseudo *R*^2^	8%	15%	10%	6%	12%	10%
Estimated probability	43%	80%	62%	58%	85%	65%

*^*a*^Reference variable. ^∗∗∗^, ^∗∗^, and ^∗^ indicate that the explanatory variables are significant at 1, 5, and 10%.*

**TABLE 5:** Estimations of wellbeing indicators: Model 2 and Model 3 by gender (Logit: dy/dx).

	**Women**			**Men**		
		
	**Good health**	**Happiness**	**Life satisfaction**	**Good health**	**Happiness**	**Life satisfaction**
**Model 2**						
UnpaidWork	0.024	–0.068^∗∗^	0.011	0.100^∗∗^	0.064^∗∗^	0.093^∗∗^
Pseudo *R*^2^	8%	15%	10%	7%	13%	11%
Estimated probability	44%	80%	62%	58%	85%	65%
**Model 3**						
UnpaidSocialAwareness	–0.052	0.095^∗^	0.001	0.046	0.097^∗∗^	0.198^∗∗^
UnpaidProfessional	0.101	0.092	0.075	0.003	0.062	0.015
UnpaidEducationLeisure	0.047	0.075	0.082	0.052	0.037	–0.034
UnpaidReligion	0.002	0.052^∗^	0.008	0.071	0.052	0.139^∗∗∗^
Pseudo *R*^2^	8%	16%	10%	7%	14%	12%
Estimated probability	44%	80%	62%	58%	85%	65%

*^*a*^We have included explanatory variables of Model 1. Estimated coefficients are robust, so we have omitted their transcription. Results are available on request to authors. ^∗∗∗^, ^∗∗^, and ^∗^ indicate that the explanatory variables are significant at 1, 5, and 10%.*

Results by gender reveal that age has a stronger impact on women than on men (three significative and relevant estimated coefficients for women versus one for men). Younger women enjoy better health (dy/dx: 0.077^∗^) but are the less satisfied with life (dy/dx: 0.061^∗∗^); women aged from 66 to 70 years old are the happiest (dy/dx: −0.073^∗^ for women aged from 71 to 80 years old). Men aged from 71 to 75 years old are happier (dy/dx: 0.073^∗^) than men from 66 to 70 years old. Female happiness is more strongly affected by civil status than male happiness (three significative and relevant estimated coefficients for women versus one for men). Unemployed women report better health (dy/dx: 0.326^∗∗^) than retired women; unemployed men are more satisfied with life (dy/dx: 0.160^∗^) than those that are retired. Male workers have better health (dy/dx: 0.089^∗^) than retired men. Education has a stronger impact on women’s health (dy/dx: 0.186^∗∗∗^ and 0.224^∗∗^ for secondary studies and tertiary studies) than on men’s health (dy/dx: 0.179^∗∗^ for tertiary studies), while the opposite is true for happiness (dy/dx: 0.120^∗∗∗^ and 0.161^∗∗^ for secondary studies and tertiary studies for men). Income level has a stronger impact on women’s self-reported health (dy/dx: 0.128^∗∗∗^ and 0.295^∗∗∗^ for middle income and high income) than on men’s health (dy/dx: 0.110^∗∗∗^ for middle income). Men and women with a medium-level income are happier (dy/dx: 0.109^∗∗∗^ for middle income for women and dy/dx: 0.069^∗∗^ for middle income for men) and more satisfied with life (dy/dx: 0.101^∗∗∗^ for middle income for women and dy/dx: 0.123^∗∗∗^ for middle income for men) than people with a low income. The average national salary has a high and positive impact on female life satisfaction (dy/dx: 1.420^∗∗^). Public expenditure on old age issues improves men’s opinions of their own health (dy/dx: 0.231^∗∗∗^) and happiness (dy/dx: 0.129^∗∗^) and women’s happiness (dy/dx: 0.340^∗∗^).

Voluntary activity improves men’s wellbeing (dy/dx: 0.100^∗∗^, 0.064^∗∗^, and 0.093^∗∗^ for good health, happiness, and life satisfaction), but decreases women’s levels of happiness (dy/dx: −0.068^∗∗^). Being a volunteer in activities related to social awareness is positive for the happiness of both genders (dy/dx: 0.095^∗^ and 0.097^∗∗^ for women and men) and life satisfaction for men (dy/dx: 0.198^∗∗^). Volunteering in activities related to religious issues is positive for female happiness (dy/dx: 0.052^∗^) and male life satisfaction (dy/dx: 0.139^∗∗∗^). Meanwhile, it is shown in Table 3 that social awareness volunteering has a negative impact on happiness, while the estimated coefficients in Table 4 report a positive correlation. This change of sign is caused by the introduction of macro variables. For example, in Table 5, we have controlled results taking into account national public expenditures on old age, and we observe that higher expenditures have a positive impact on happiness. People volunteer because they want to feel useful but also because they want to improve situations that they might consider undesirable. To a large degree, volunteerism behaves as an alternative agent for the government. As one goal of public expenditure on social issues is to amend precarious situations, the introduction of these variables reinforces the fact that volunteering is engaged in for the pleasure of doing it. Lastly, the impact of intrinsic and extrinsic motivations is the sum of two excluding categories (intrinsic motivation: social awareness and religion; extrinsic motivation: professional and leisure activities). We have not calculated them because the lack of confidence level for certain categories gives greater volatility to their estimated results.

## Discussion

The primary aim of this work was to provide an approximation to the determinants of wellbeing among the older adults of Spain and Latin America. Our results suggest that socioeconomic characteristics (especially income and level of education) are important predictors of wellbeing. Public policies to reduce poverty by improving training and work expectations are therefore recommended. Gender analysis reveals that women would especially benefit from these types of initiatives.

Volunteering seems to improve life satisfaction and health but not happiness (H1). Being a volunteer in activities related to social awareness has a negative impact on happiness, whereas religion-based voluntary activity seems to improve life satisfaction (H2). While economic factors have a stronger impact on female wellbeing than on male wellbeing, voluntary activities have a stronger impact on male wellbeing than female wellbeing (H3). Lastly, when controlled for socioeconomic determinants, Spaniards are more likely to have a good state of health than Mexicans and Chileans, but Mexicans are more likely to be happy than Spaniards. These results remain only for women when repeating estimations by gender (H4).

Observational evidence suggests that volunteering can benefit health and life expectancy, even though the causal mechanisms remain unclear. This information could be used in the design of a political agenda that promotes health and wellbeing (Jenkinson et al., 2013).

Despite the fact that participation in voluntary activities generates a range of benefits for all those involved, awareness of volunteering options is still limited. There are probably many older adults who do not volunteer as they simply do not know that opportunities exist. Given the synergy that arises from social cooperation and wellbeing, volunteerism should be given much more attention and consideration; it should be recognized as a key element in the development of social cohesion and given much more visibility in society (Deloitte Consulting, 2012). Barriers, such as limited economic resources or mobility problems, should be removed to facilitate and encourage the participation of our older generations in both formal and informal voluntary projects (Martinez et al., 2011; Pozzi et al., 2014).

Even though estimated parameters of country dummy variables are in the argumentation line of descriptive statistics about wellbeing by country, there is no doubt that controlling estimations by explanatory factors reduces the importance of national gaps. The low number of countries and survey waves does not allow us to generalize our results. Increasing the number of countries and socioeconomic variables will give us more information about whether national differences remain. Social participation of older people is the result of personal decisions, but it is influenced by other factors and barriers that we should take into account (Majón-Valpuesta et al., 2016).

From a technical perspective, the cross-sectional design might include causal inferences (thus, whether voluntary activities have an effect on subjective wellbeing or vice versa, or whether some third variable—such as living conditions or personality traits—has an effect on both). Longitudinal and multilevel models could also enrich the methodological design of this research (Ferring and Boll, 2010).

From a psychological perspective, one limitation of this research is the lack of open questions addressed at understanding the self-evaluation of the volunteer experience (Morrow-Howell, 2010). Open questions about volunteering would have clarified whether volunteering improves wellbeing and happiness because this behavior is a source of pleasure (hedonistic hypothesis: Ryan and Deci, 2001), or a source of fulfillment (eudemonic hypothesis: McMahan and Renken, 2011) or both (Diener, 2006, 2009).

The link between volunteering and wellbeing is complex and requires further research. Future studies should include the frequency and length of social activities (Takeuchi et al., 2013), reasons for volunteering in each category (Pi et al., 2014), attitudes toward volunteering (Pettigrew et al., 2015), and the organizational perspective (Ishikawa et al., 2016).

According to the literature review, volunteering shows positive psychosocial effects in fields such as social connectedness and social networks, commitment in social activities, and social meaning of life that stimulate protective mechanisms in mental and physical health (Thoits and Hewitt, 2001; Seligman, 2002; Kaneda et al., 2011; Fiorillo and Nappo, 2014; Coll-Planas et al., 2017). Social participation through volunteering allows older people to contribute to the common good, enhancing social improvements and ensuring continuity among generations. Volunteering should be encouraging throughout the individual lifecycle to promote wellbeing in old age. Organizations should be able to provide opportunities that allow volunteers to satisfy their primary motivations because, by doing so, benefits will potentially increase for all (Cornelis et al., 2013).

## Data Availability Statement

Publicly available datasets were analyzed in this study. This data can be found here: micro-data from the World Value Survey (1994–1998; 2005–2009, and 2010–2014).

## Ethics Statement

Ethics for this protocol was not required, as all of the data analyzed was obtained from existing publications.

## Author Contributions

MS-G, AG-L, and MG-L contributed to the conception and design of the study and wrote sections of the manuscript. AG-L organized the database and performed the statistical analysis. MS-G and AG-L wrote the first draft of the manuscript. All authors contributed to the manuscript revision and read and approved the submitted version.

## Conflict of Interest

The authors declare that the research was conducted in the absence of any commercial or financial relationships that could be construed as a potential conflict of interest.

## Footnotes

1 http://www.worldvaluessurvey.org/wvs.jsp

2 http://www.oecd.org/els/health-systems/health-data.htm

## References

[B1] Ahern M. M. Hendryx M. (2008). Community participation and the emergence of late-life depressive symptoms: differences between women and men. *J. Womens Health* 17 1463–1470. 10.1089/jwh.2007.0752 18945207

[B2] Amendola A. Garofalo M. R. Nese A. (2011). Versus poverty traps is the third sector an emerging economic institution? Social preferences. *Nonprofit Volunt. Sect. Q.* 40 850–872. 10.1177/0899764010371232

[B3] Ariza-Montes A. Tirado-Valencia P. Fernández-Rodríguez V. Leal-Rodríguez A. (2017). Volunteering by elders: a question of values? *Serv. Ind. J.* 37 685–702. 10.1080/02642069.2017.1298095 9216553

[B4] Barron J. S. Tan E. J. Yu Q. Song M. McGill S. Fried L. P. (2009). Potential for Intensive Volunteering to Promote the Health of Older Adults in Fair Health. *J. Urban Health* 86 641–653. 10.1007/s11524-009-9353-8 19488860 PMC2704275

[B5] Bartels M. Boomsma D. I. (2009). Born to be Happy? The Etiology of Subjective Well-Being. *Behav. Genet.* 39 605–615. 10.1007/s10519-009-9294-9298 19728071 PMC2780680

[B6] Binder M. (2015). Volunteering and life satisfaction: a closer look at the hypothesis that volunteering more strongly benefits the unhappy. *Appl. Econ. Lett.* 22 874–885. 10.1080/13504851.2014.985364

[B7] Binder M. Freytag A. (2013). Volunteering, subjective well-being and public policy. *J. Econ. Psychol.* 34 97–119. 10.1016/j.joep.2012.11.008

[B8] Bloomberg L. (2014). *Most Efficient Health Care 2014.* Available at: http://www.bloomberg.com/visual-data/best-and-worst//most-efficient-health-care-2014-countries. (accessed June 23, 2015)

[B9] Borgonovi F. (2008). Doing well by doing good. The relationship between formal volunteering and self-reported health and happiness. *Soc. Sci. Med.* 66 2321–2334. 10.1016/j.socscimed.2008.01.011 18321629

[B10] Brett C. E. Gow A. J. Corley J. Pattie A. Starr J. M. Deary I. J. (2012). Psychosocial factors and health as determinants of quality of life in community-dwelling older adults. *Qual. Life Res.* 21 505–516. 10.1007/s11136-011-9951-2 21706382

[B11] Bunout D. Osorio P. Barrera G. Torrejon M. J. Meersohn C. Anigstein M. S. (2012). Quality of life of older Chilean people living in metropolitan Santiago, Chile: influence of socioeconomic status. *Ageing Res.* 4 15–18.

[B12] Burr J. A. Tavares J. Mutchler J. E. (2011). Volunteering and hypertension risk in later life. *J. Aging Health* 23 24–51. 10.1177/0898264310388272 20971920

[B13] Chen H. Morrow-Howell N. (2015). Antecedents and Outcomes of Older Adults’ Motivations to Volunteer With Experience Corps. *Res. Hum. Dev.* 12 118–132. 10.1080/15427609.2015.1010352

[B14] Chen L. M. (2013). Senior Volunteerism in Japan: A Policy Perspective. *Ageing Int.* 38 97–107. 10.1007/s12126-012-9168-x

[B15] Cheng G. Chan A. (2018). Volunteering Among Older Middle-Aged Singaporeans: A Latent Class Analysis. *Innov. Aging* 2:565 10.1093/geroni/igy023.2091

[B16] Cocca-Bates K. C. Neal- Boylan L. (2011). Retired RNs: Perceptions of Volunteering. *Geriatr. Nurs.* 32 96–105. 10.1016/j.gerinurse.2010.11.003 21227547

[B17] Coll-Planas L. Gomez G. D. Bonilla P. Masat T. Puig T. Monteserin R. (2017). Promoting social capital to alleviate loneliness and improve health among older people in Spain. *Health Soc. Care Community* 25 145–157. 10.1111/hsc.12284 26427604

[B18] Cornelis I. Van Hiel A. De Cremer D. (2013). Volunteer work in youth organizations: predicting distinct aspects of volunteering behavior from self- and other-oriented motives. *J. Appl. Soc. Psychol.* 43 456–466. 10.1111/j.1559-1816.2013.01029.x

[B19] Deloitte Consulting, (2012). *Evaluation of the European Year of Volunteering 2011.* Available at: http://ec.europa.eu/dgs/communication/about/evaluation. (accessed June 23, 2015).

[B20] Diener E. (2006). Guidelines for national indicators of subjective well-being an ill-being. *Appl. Res. in Qual. Life* 1 151–157. 10.1007/s11482-006-9007-x

[B21] Diener E. (2009). *The Science of Well-Being.* New York, NY: Springer.

[B22] Diener E. Kesebir P. Lucas R. (2008). Benefit of Accounts of Well-being - for Societies and for Psychological Science. *Appl. Psychol.* 57 37–53. 10.1111/j.1745-6916.2007.00030.x 26151920

[B23] Doyal L. (2000). Gender equity in health: debates and dilemmas. *Soci. Sci. Med.* 51 931–939. 10.1016/s0277-9536(00)00072-1 10972436

[B24] Einolf C. J. (2011). Gender Differences in the Correlates of Volunteering and Charitable Giving. *Nonprofit Volunt. Sect. Q.* 40 1092–1112. 10.1177/0899764010385949

[B25] Estrada-Fernandez M. E. Gil-Lacruz A. I. Gil-Lacruz M. Viñas-Lopez A. (2017). La dependencia: efectos en la salud familiar. *Atención Primaria* 3 27–45. 10.1016/j.aprim.2016.12.007 PMC6836982 28431761

[B26] Ferring D. Boll T. (2010). “Subjective Well-being in Older Adults: Current State and Gaps of Research,” in *Ageing, Health and Pensions in Europe*, eds Bovenberg L. van Soest A. Zaidi A. (London: Palgrave Macmillan).

[B27] Fiorillo D. Nappo N. (2014). *Formal and Informal Volunteering and Health Across European Countries*. *MPRA Paper No. 54130*. Available at: https://mpra.ub.uni-muenchen.de/54130/1/MPRA_paper_54130.pdf (accessed June 23, 2015).

[B28] Fiorillo D. Sabatini F. (2011). Quality and quantity: the role of social interactions in individual health. *Soc. Sci. Med.* 73 1644–1652. 10.1016/j.socscimed.2011.09.007 22001229

[B29] Fischer L. Schaffer K. (1993). *Older Volunteers.* Newbury Park, CA: Sage.

[B30] Fothergill K. E. Ensminger M. E. Robertson J. Green K. M. Thorpe R. J. Juon H. S. (2011). Effects of social integration on health: A prospective study of community engagement among African American women. *Soc. Sci. Med.* 72 291–298. 10.1016/j.socscimed.2010.10.024 21131117 PMC3031118

[B31] Frenk J. Gómez-Dantés O. Knaul F. M. (2009). The democratization of health in Mexico: financial innovations for universal coverage. *Bull. World Health Org.* 87 542–548. 10.2471/blt.08.053199 19649369 PMC2704036

[B32] Gallo P. Gené-Badía J. (2013). Cuts drive health system reforms in Spain. *Health Policy* 113 1–7. 10.1016/j.healthpol.2013.06.016 24035010

[B33] Gil-Lacruz A. I. Gil-Lacruz M. Leeder S. (2015). Women and smoking – prices and health warming messages: evidence from Spain. *Addict. Behav.* 45 294–300. 10.1016/j.addbeh.2015.01.016 25770976

[B34] Gil-Lacruz A. I. Marcuello C. (2013). Voluntary Work in Europe: Comparative Analysis Among Countries and Welfare Systems. *Soc. Indic. Res.* 114 371–382. 10.1007/s11205-012-0150-5

[B35] Guven C. (2011). Are happier people better citizens? *Kyklos* 64 178–192. 10.1111/j.1467-6435.2011.00501.x 14585512

[B36] Harlow R. E. Cantor N. (1996). Still participating after all these years: a study of life task participation in later life. *J. Pers. Soc. Psychol.* 71 1235–1249. 10.1037//0022-3514.71.6.1235 8979389

[B37] Harvey S. J. (2011). Is the just man a happy man? An empirical study of the relationship between ethics and subjective well-being. *Kyklos* 64 193–212. 10.1111/j.1467-6435.2011.00502.x

[B38] Heist H. D. Cnaan R. A. Lough B. J. (2019). Determinants of serving a mission: Senior volunteering among Latter-Day Saints. *Psychol. Religion Spiritual.* 10.1037/rel0000246

[B39] House J. S. Landis K. R. Umberson D. (1988). Social relationships and health. *Science* 241 540–545. 3399889 10.1126/science.3399889

[B40] Ilesanmi O. O. (2009). What is cross-cultural research. *Int. J. Psychol. Stud.* 1 82–96.

[B41] Ishikawa Y. Kondo N. Kondo K. Saito T. Hayashi H. Kawachi I. (2016). Social participation and mortality: does social position in civic groups matter?. *BMC Public Health* 16:394 10.1186/s12889-016-3082-3081 PMC4866293 27175729

[B42] Jenkinson C. E. Dickens A. P. Jones K. Thompson-Coon J. Taylor R. S. Rogers M. (2013). Is volunteering a public health intervention? A systematic review and meta-analysis of the health and survival of volunteers. *BMC Public Health* 13:773. 10.1186/1471-2458-13-773 23968220 PMC3766013

[B43] Kaneda T. Lee M. Pollard K. (2011). *SCL/PRB Index of Well-Being in Older Populations, Final Report, Global Aging and Monitoring Project, Stanford Center on Longevity and the Population Reference Bureau.* Washington, D.C: Population Reference Bureau.

[B44] Kwok Y. Y. Chui W. H. Wong L. P. (2013). Need Satisfaction Mechanism Linking Volunteer Motivation and Life Satisfaction: A Mediation Study of Volunteers Subjective Well-Being. *Soc. Indic. Res.* 114 1315–1329. 10.1007/s11205-012-0204-8

[B45] Majón-Valpuesta D. Ramos P. Pérez-Salanova M. (2016). Claves para el análisis de la participación social en los procesos de envejecimiento de la generación baby boom. *Psicoperspectivas* 15 53–63.

[B46] Mannino C. A. Snyder M. Omoto A. M. (2011). “Why do people get involved? Motivations for volunteerism and other forms of social action,” in *Social Motivation*, ed. David D. (New York, NY: Psychology Press), 127–146.

[B47] Martinez I. L. Crooks D. Kim K. S. Tanner E. (2011). Invisible civic engagement among older adults: valuing the contributions of informal volunteering. *J. Cross Cult. Gerontol.* 26 23–37. 10.1007/s10823-011-9137-y 21243418

[B48] McMahan E. A. Renken M. D. (2011). Eudaimonic conceptions of well-being, meaning in life, and self-reported well-being: Initial test of a mediational model. *Pers. Individ. Dif.* 51 589–594. 10.1016/j.paid.2011.05.020

[B49] Meier S. Stutzer A. (2004). Is Volunteering Rewarding in Itself? *Economica* 75 39–59.

[B50] Missoni E. Solimano G. (2010). *Towards universal health coverage: the Chilean experience. World Health Report, Background paper, 4, World Health Organization.* Available at: http://www.who.int/healthsystems/topics/financing/healthreport/4Chile.pdf (accessed June 23, 2015)

[B51] Moen P. Dempster-McClain D. Williams R. (1992). Successful aging: A life course perspective on women’s multiple roles and health. *Am. J. Sociol.* 97 1612–1638. 10.1086/229941

[B52] Morrow-Howell N. (2010). Volunteering in later life: research frontiers. *J. Gerontol. Psychol. Soc. Sci.* 65 461–469. 10.1093/geronb/gbq024 20400498

[B53] Moss N. E. (2002). Gender equity and socioeconomic inequality: a framework for the patterning of women’s health. *Soc. Sci. Med.* 54 649–661. 10.1016/s0277-9536(01)00115-0 11999484

[B54] Musick M. Herzog A. House J. (1999). Volunteering and mortality among older adults: Findings from a national sample. *J. Gerontol.* 54 173–180. 10.1093/geronb/54b.3.s173 10363048

[B55] Okun M. A. August K. J. Rook K. S. Newsom J. T. (2010). Does volunteering moderate the relation between functional limitations and mortality? *Soc. Sci. Med.* 71 1662–1668. 10.1016/j.socscimed.2010.07.034 20864238 PMC2975672

[B56] Oman D. Thoresen C. McMahon K. (1999). Volunteerism and mortality among older adults: Findings from a national sample. *J. Health Psychol.* 4 301–316. 22021599 10.1177/135910539900400301

[B57] Pettigrew S. Jongenelis M. Newton R. U. Warburton J. Jackson B. (2015). Research protocol for a randomized controlled trial of the health effects of volunteering for seniors. *Health Qual. f Life Outcomes* 13:74. 10.1186/s12955-015-0263-z 26040633 PMC4453438

[B58] Pi L. L. Lin Y. H. Chen C. Y. Chiu J. C. Chen Y. M. (2014). Serious leisure, motivation to volunteer and subjective well-being of volunteers in recreational events. *Soc. Indic. Res.* 119 1485–1494. 10.1007/s11205-013-0562-x

[B59] Pinquart M. Sörensen S. (2000). Influences of socieconomic status, social networks, and competence on subjective well-being in later life: a meta-analysis. *Psychol. Aging* 15 187–224. 10.1037//0882-7974.15.2.187 10879576

[B60] Population Reference Bureau, (2014). *Rate of Natural Increase.* Available at: http://www.prb.org/datafinder/topic/rankings.aspx?ind=16. (accessed June 23, 2015)

[B61] Post S. G. (2005). Altruism, happiness, and health: It’s good to be good. *Int. J. Behav. Med.* 12 66–77. 10.1207/s15327558ijbm1202_4 15901215

[B62] Pozzi M. Marta E. Marzana D. Gozzoli C. Ruggieri R. A. (2014). The Effect of Psychological Sense of Community on the Psychological Well-Being in Older Volunteers. *Eur. J. Psychol.* 10 598–612. 10.5964/ejop.v10i4.773

[B63] Pushkar D. Chaikelson J. Conway M. Etezadi J. Giannopoulus C. Li K. (2010). Testing Continuity and Activity Variables as Predictors of Positive and Negative Affect in Retirement. *J. Gerontol. B Psychol. Sci. Soc. Sci.* 65 42–49. 10.1093/geronb/gbp079 19875749

[B64] Rogers R. (1996). The effects of family composition, health, and social support linkages on mortality. *J. Health Soc. Behav.* 37 326–338. 8997888

[B65] Ronel N. (2006). When good overcomes bad: The impact of volunteers on those they help. *Hum. Relat.* 59 1133–1153. 10.1177/0018726706068802

[B66] Ruiz-Aranda D. Extremera N. Pineda-Galán C. (2014). Emotional intelligence, life satisfaction and subjective happiness in female student health professionals: the mediating effect of perceived stress. *J. Psychiatr. Ment. Health Nurs.* 21 106–113. 10.1111/jpm.12052 23578272

[B67] Russell A. R. Nyame-Mensah A. de Wit A. Handy F. (2019). Volunteering and Wellbeing Among Ageing Adults: A Longitudinal Analysis. *Voluntas* 30 115–128. 10.1007/s11266-018-0041-8

[B68] Ryan R. M. Deci E. L. (2001). On happiness and human potentials: A review of research on hedonic and eudaimonic well-being. *Annu. Rev. Psychol.* 52 141–166. 10.1146/annurev.psych.52.1.141 11148302

[B69] Sabatini F. (2008). Social capital and the quality of economic development. *Kyklos* 61 466–499.

[B70] Sabin E. P. (1993). Social relationships and mortality among the elderly. *J. Appl. Gerontol.* 12 44–60. 10.1177/073346489301200105

[B71] Salvador-Carulla L. Lucas-Carrasco R. Ayuso-Mateos J. L. Miret M. (2014). Use of terms “Wellbeing” and “Quality of Life” in health sciences: A conceptual framework. *Eur. J. Psychiatry* 28 50–65. 10.4321/s0213-61632014000100005

[B72] Sarason S. B. (1986). The emergence of a conceptual center. *J. Community Psychol.* 14 405–407. 10.1002/1520-6629(198610)14:4<405::aid-jcop2290140409>3.0.co;2-8

[B73] Sardinha B. (2010). *The Economics of the Volunteering Decision.* Unpublished doctoral thesis, Faculdade de Economia, Évora.

[B74] Seligman M. E. P. (2002). *Authentic Happiness: Using the New Positive Psychology to Realise Your Potential For Lasting Fulfilment.* New York, NY: Free Press.

[B75] South J. Southby K. James M. Tree D. Buck D. (2016). Exploring the links between volunteering, health and inequalities – is this a public health issue? *Eur. J. Public Health* 26:ckw171.013 10.1093/eurpub/ckw171.013

[B76] Stephan P. (1991). Relationships among market work, work aspirations and volunteering: The case of retired women. *Nonprofit Volunt. Sect. Q.* 20 225–236. 10.1177/089976409102000208

[B77] Stephens C. Breheny M. Mansvelt J. (2015). Volunteering as Reciprocity: beneficial and Harmful Effects of Social Policies to Encourage Contribution in Older Age. *J. Aging Stud.* 33 22–27. 10.1016/j.jaging.2015.02.003 25841726

[B78] Stukas A. A. Hoye R. Nicholson M. Brown K. M. Aisbett L. (2016). Motivations to Volunteer and Their Associations With Volunteers’ Well-Being. *Nonprofit Volunt. Sect. Q.* 45 112–132. 10.1177/0899764014561122 9464092

[B79] Takeuchi K. Aida J. Kondo K. Osaka K. (2013). Social participation and dental health status among older Japanese adults: a population-based cross-sectional study. *PloS One* 8:e61741. 10.1371/journal.pone.00617141 23613921 PMC3629217

[B80] Taylor R. F. (2004). Extending conceptual boundaries: Work, voluntary work and employment. *Work Employ. Soc.* 18 29–49. 10.1177/0950017004040761

[B81] Thoits P. A. Hewitt L. N. (2001). Volunteer work and well-being. *J. Health Soc. Behav.* 42 115–131. 11467248

[B82] Urzúa A. Bravo M. Ogalde M. Vargas C. (2011). Factores vinculados a la calidad de vida en la adultez mayor. *Revista Medicina Chile* 139 1006–1014. 10.4067/s0034-98872011000800005 22215330

[B83] Vagetti G. C. Barbosa V. C. Moreira N. B. de Oliveira V. D. Mazzardo O. de Campos W. (2015). The association between physical activity and quality of life domains among older women. *J. Aging Phys. Act.* 23 524–533. 10.1123/japa.2013-0070 25415389

[B84] Vargas V. Poblete S. (2008). Health prioritization: the case of Chile. *Health Aff.* 27 782–792. 10.1377/hlthaff.27.3.782 18474972

[B85] Wei I. I. Virning B. A. John D. A. Morgan R. O. (2006). Using a Spanish surname match to improve identification of Hispanic women in Medicare administrative data. *Health Serv. Res.* 41 1469–1481. 16899019 10.1111/j.1475-6773.2006.00550.x PMC1797094

[B86] WHO, (2012). *1st World Congress on Healthy Ageing. 19th-22nd March 2012, Kuala Lumpur Convention Centre, Kuala Lumpur, Malaysia.* Available at: http://www.healthyageingcongress.com/#sthash.Uhk0FOHk.dpuf (accessed June 23, 2015)

[B87] Wilson J. Musick M. (1999). The Effects of Volunteering on the Volunteer. *Law Contemp. Probl.* 62 141–168.

[B88] Wong R. Espinoza I. Palloni A. (2010). Adultos mayores mexicanos en un contexto socioeconómico amplio: salud y envejecimiento. *Salud Pública México* 49 436–447. 10.1590/s0036-36342007001000002 17724516

[B89] World Values Survey Association, (2017). *World Values Survey.* Available at: http://www.worldvaluessurvey.org/wvs.jsp (accessed December 26, 2017)

